# Supplementary material for: Complex‐centric proteome profiling by SEC‐SWATH‐MS
Source: Mol Syst Biol. 2019 Jan 14;15(1):e8438. doi: 10.15252/msb.20188438 (PMC6346213; doi:10.15252/msb.20188438)
Supplement: Supplementary file 8 — Dataset EV7 [file MSB-15-e8438-s008.zip › feature_plots_string/Q9Y4Z0.pdf]

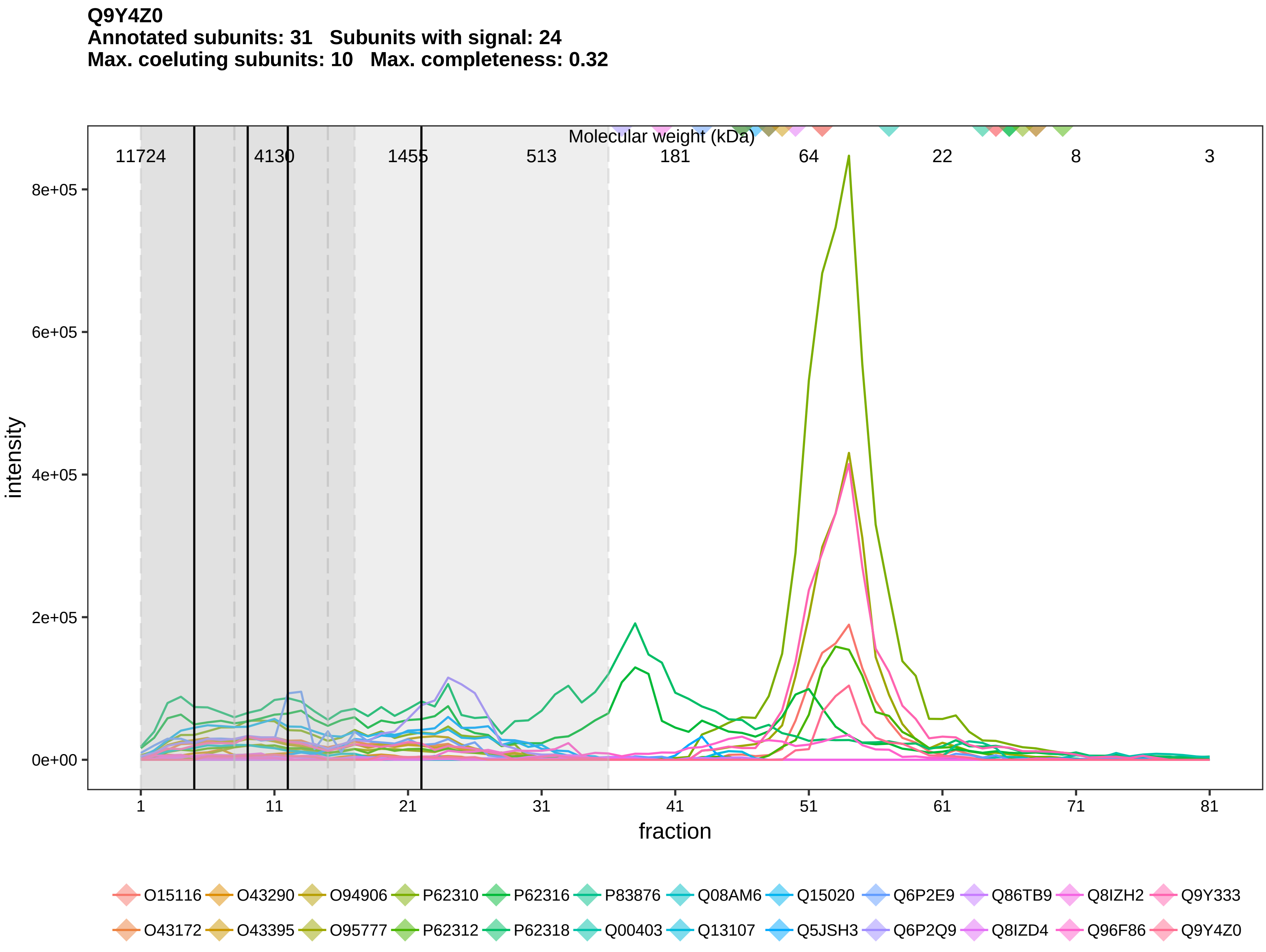
Q9Y4Z0
Annotated subunits: 31 Subunits with signal: 24
Max. coeluting subunits: 10 Max. completeness: 0.32
Molecular weight (kDa)
11724
4130
1455
513
181
64
22
8
3
8e+05
6e+05
4e+05
2e+05
0e+00
intensity
1
11
21
31
41
51
61
71
81
fraction
O15116
O43290
O94906
P62310
P62316
P83876
Q08AM6
Q15020
Q6P2E9
Q86TB9
Q8IZH2
Q9Y333
O43172
O43395
O95777
P62312
P62318
Q00403
Q13107
Q5JSH3
Q6P2Q9
Q8IZD4
Q96F86
Q9Y4Z0
